# Lattice dynamics in CePd_2_Al_2_ and LaPd_2_Al_2_

**DOI:** 10.1038/s41598-021-99904-7

**Published:** 2021-10-22

**Authors:** Petr Doležal, Petr Cejpek, Satoshi Tsutsui, Koji Kaneko, Dominik Legut, Karel Carva, Pavel Javorský

**Affiliations:** 1 Faculty of Mathematics and Physics, Department of Condensed Matter Physics, Charles University, Ke Karlovu 5, 121 16 Prague 2, Czech Republic; 2 Japan Synchrotron Radiation Research Institute (JASRI), SPring-8, Sayo, Hyogo, 679-5198 Japan; 3 Institute of Quantum Beam Science, Graduate School of Science and Engineering, Ibaraki University, Hitachi Ibaraki, 316-8511 Japan; 4 Materials Science Research Center, Japan Atomic Energy Agency, Tokai Ibaraki, 319-1195 Japan; 5 IT4Innovations, VSB-Technical University of Ostrava, 17. listopadu 2172/15, 708 00 Ostrava, Czech Republic

**Keywords:** Condensed-matter physics, Magnetic properties and materials

## Abstract

The interaction between phonons and 4f electrons, which is forming a new quantum state (quasi-bound state) beyond Born-Oppenheimer approximation, is very prominent and lattice dynamics plays here a key role. There is only a small number of compounds in which the experimental observation suggest such a scenario. One of these compounds is CePd_2_Al_2_. Here the study of phonon dispersion curves of (Ce,La)Pd_2_Al_2_ at 1.5, 7.5, 80 and 300 K is presented. The inelastic X-ray scattering technique was used for mapping the phonon modes at X and Z points as well as in Λ and Δ directions, where the symmetry analysis of phonon modes was performed. The measured spectra are compared with the theoretical calculation, showing very good agreement. The measurements were performed in several Brillouin zones allowing the reconstruction of phonon dispersion curves. The results are discussed with respect to the magneto-elastic interaction and are compared with other cerium compounds. The phonon mode symmetry A_1g_ was found to be unaffected by the interaction, which is in contrast to previous assumptions.

## Introduction

Physical properties of intermetallic compounds are driven by their electronic properties and lattice dynamics, but usually their impact is treated separately. This is also the case of magnetism in rare earth compounds. The localised character of 4f electrons and their geometrical arrangement in the crystal lattice determine the magnetic behaviour and the lattice dynamics is usually not considered. This approach is based on the Born-Oppenheimer approximation, where the difference of several orders of magnitude in mass of atomic nucleus and electrons typically justify this attitude.

In standard crystal electrical field (CEF) theory the rare earth ion with full rotation symmetry is placed into a static crystal lattice, which decreases degrees of freedom and reduces the degeneracy of a ground state depending on the point symmetry of a given site in the crystal lattice. It was shown, that this approach cannot explain the magnetic excitation spectra measured in several compounds, e.g. in pyrochlores Tb_2_Ti_2_O_7_^1^, Ho_2_Ti_2_O_7_^2^, intermetallics CeAl_2_^3^, PrNi_2_^4^, CePd_2_Al_2_^5^, CeCuAl_3_^6^ and recently in CeAuAl_3_^7^. The Ce-based intermetallics have a very prominent position with a relatively small number of expected CEF excitations. The oxidation number of the Ce ion in the materials listed above is 3+, implying total angular momentum $J=\frac{5}{2}$ and a six fold degenerated ground state. The half integer moment requires at least two fold degeneracy even in triclinic site symmetry in the crystal lattice. This simply restricts the number of magnetic excitations from the ground state to two. The higher number of observed magnetic excitations in the spectra than two is very peculiar. One of the possible explanations of such behaviour was found in the coupling of lattice dynamics and 4f electronic states, which is usually neglected in the Born-Oppenheimer approximation as mentioned above. This would lead to the formation of a new quantum state^8^ and opens new questions about its origin and conditions which have to be fulfilled. All these aspects make these compounds very interesting especially in the case when an X-ray free electron laser opens new possibilities to study the lattice dynamics.

The CeAl_2_ crystallises in a cubic structure of type MgCu_2_ Laves phase^9^. The site symmetry of the Ce ion is also cubic, therefore only two levels $\Gamma _7^2$ and $\Gamma _8^4$ (based on CEF theory) and one excitation from ground state are expected. Surprisingly, two excitations from the doublet $\Gamma _7^2$ have been observed by inelastic neutron scattering^3^. Simultaneously the study of phonon dispersion curves by inelastic neutron scattering shows a softening and broadening of the $\Gamma _{25}^{\prime }$ phonon mode and symmetry related phonon branches in the $\Lambda$ direction^10^. Both observed features were explained by the existence of a quasi-bound state between 4f electrons and phonons^8^. The interaction term in the model Hamiltonian uses the crystal electrical field operator with the same transformation properties as the $\Gamma _{25}^{\prime }$ mode^8^. This shows how the knowledge of phonon dispersion curves play a key role in search for the model Hamiltonian. Without direct observation of the influence of a quasi-bound state on phonons, the presence of the quasi-bound state remains still an open question.

The experimental studies in CeCuAl_3_^6^, CePd_2_Al_2_^5^ and CeAuAl_3_^7^ propose the existence of a bound state, but its signature was found mainly in the magnetic excitations spectra. The study of phonon dispersion curves is still missing with some exception for CeAuAl_3_ and CeCuAl_3_^7^^11^. The study of low energy phonon dispersion curves (mainly acoustic) on CeAuAl_3_^7^ shows the anticrossing of a non-dispersive magnetic excitation and an acoustic phonon branch, which was not observed before. The softening or broadening as known for CeAl_2_ was not found in the measured energy interval^7^.

Our present study of single crystal CePd_2_Al_2_ and LaPd_2_Al_2_ is focused on phonon dispersion curves studied by inelastic X-ray scattering. The non-magnetic La homologue is used as a reference sample, because the La ion have no 4f electrons and therefore the bound state between 4f electrons and phonons is not present here. We performed the symmetry analysis of phonon modes at $\Gamma$ and X points and in the $\Delta$ and $\Lambda$ directions, which were calculated in^12^. Based on the symmetry analysis we focused on selective phonon modes, especially with symmetry used in the model Hamiltonian^5^ and compare it with the experimental data. The results are discussed in terms of a possible appearance of a quasi bound state in these Ce based intermetallics.

## Methods

Single crystals of CePd_2_Al_2_ and LaPd_2_Al_2_ were prepared by the Czochralski method. As these compounds are incongruently melting, the procedure described in^13^ was adopted. The starting compositions of the melted precursors were 22:41.1:36.9 (Ce:Pd:Al) and 22:39:39 (La:Pd:Al). The phase purity in the prepared ingots was checked by X-ray powder diffraction performed on Bruker D8 Advance diffractometer in Bragg-Brentano geometry with variable slits. The patterns of both Ce and La homologs were fit by a CaBe_2_Ge_2_ structural model using the Rietveld method with resulting lattice parameters $a = b = 4.4112(3)$ Å, $c = 9.8749(9)$ Å  and $a = b = 4.439(1)$ Å, $c = 9.915(3)$ Å, respectively. The chemical composition was analysed by Energy-dispersive X-ray analysis and phase purity was also confirmed by backscattered electrons imaging performed on an electron microscope TESCAN, type Mira I LMH. The resulting composition of samples were 18:41:41 and 20:40:40 atomic precent of Ce:Pd:Al and La:Pd:Al. The measured chemical composition corresponds to ideal one within 2 % experimental error. The sample sizes of Ce and La based samples were 1 x 0.6 x 0.6 mm and 1 x 0.8 x 0.4 mm, respectively. The single crystalline samples were oriented by Laue method using a Laue X-ray Imaging System 20041209 SY Issue 8 (Photonic Science) with CCD camera (1220-1800 pixels) and air-cooled X-ray tube (30 kV, 300 μA). The beam size 0.5 mm^2^ used for Laue method was smaller than the sample. A light-polarized microscopy was used to confirm the single-grain nature of the samples.

The previous study of polycrystalline (Ce,La)Pd_2_Al_2_ samples reveals the presence of a structural phase transition to a lower symmetry orthorhombic structure described by the *Cmme* (67) space group^5,14^. The temperature of the structural phase transitions, $T_{str}$, in prepared single crystals was checked by low temperature X-ray powder diffraction showing that the structural transitions are shifted to lower temperatures in comparison to the polycrystalline samples, $T_{str} \approxeq 6$ K for CePd_2_Al_2_ and $T_{str} \approxeq 60$ K for LaPd_2_Al_2_. In polycrystalline samples the transition temperatures were around 13.5 K (11 K) and 91.5 K (84 K)^5^(^14^) for Ce and La samples, respectively. Details of the low temperature diffraction are presented in Supplementary information.

The inelastic X-ray scattering spectra were measured at the BL35XU beamline at the SPring-8 synchrotron (Japan). The high order 11 11 11 diffraction from a Si crystal and energy of 21.747 keV of the beam were used to ensure the energy resolution of 1.5 meV. The beam size was $\approx 50$ μm. Si crystals were also used as analyser and their lattice parameters were controlled by a temperature, which allows the selection of the energy transfer. The detail description of BL35XU is given in^15^^16^.

Theoretical calculation of phonon dispersion curves in (Ce,La)Pd_2_Al_2_ are provided in^12^. For the analysis of inelastic spectra is necessary to know the polarisation and phase of displacement for all atoms in the primitive unit cell, which are given by eigenvectors $\mathbf{e} _{\mathbf{q}jd}$ and were calculated in^12^. The intensity of the selected phonon mode in a given Brillouin zone is proportional to dynamical structure factor, which one-phonon contribution is given by^16^:

1 $$\begin{aligned} \begin{array}{l} S\left( \mathbf{Q} , \omega \right) _{1p} = N \sum \limits _\mathbf{q } \sum \limits _{j} \left\| \sum \limits _{d} \frac{f_d(\mathbf{Q} )}{\sqrt{2M_d}} \quad e^{-W_d(\mathbf{Q} )} \quad \mathbf{Q} \cdot \mathbf{e} _{\mathbf{q}jd} \quad e^{i\mathbf{Q} \cdot \mathbf{x} _d} \right\| ^2 \delta _{(\mathbf{Q} -\mathbf{q} )\varvec{\tau }} \quad F_{\mathbf{q}j}(\omega ), \end{array} \end{aligned}$$

where *N* counts the irradiated unit cells, $\varvec{\tau }$ is the Bragg vector, $\mathbf{q}$ is the reduced momentum transfer in the first Brillouin zone, $\mathbf{Q} = \mathbf{q} + \varvec{\tau }$. The index *j* runs over phonon modes for a given $\mathbf{q}$ and *d* runs over the atoms in the primitive unit cell. The position of atom within the unit cell is given by $\mathbf{x} _d$, $M_d$ is its mass and $f_d$ is the atomic form-factor for X-rays. $F_{\mathbf{q}j}(\omega )$ corresponds to the energy resolution function. $W_d(\mathbf{Q} ))=B_d\frac{\vert |\mathbf{Q} \vert |^2}{16\pi ^2}$ is the Debye-Waller factor. For our calculations, we use the Debye-Waller factor approximated for the case of isotropic vibrations^17^ as:

2 $$\begin{aligned} B_d=\frac{6h^2}{M_dk_B\Theta _D}\left( \frac{\phi (\Theta _D/T)}{\Theta _D/T}+\frac{1}{4}\right) \end{aligned}$$

where *T* is the temperature, $\Theta _D$ is Debye temperature and *h* and $k_B$ are Planck and Boltzmann constant respectively. $\phi$ is the Debye function $\phi (x)=\frac{1}{x}\int _{0}^{x}\frac{\xi }{\text {e}^\xi -1}\text {d}\xi$.

The measurement was done in these Brillouin zones 0 1 8, 0 0 8, 0 1 12, 0 2 14, 4 0 1, 3 0 1 and 4 0 0. This selection was chosen to cover a wide range of phonon branches with special attention to the possible separation of mode intensities during the data analysis based on the performed simulation given by Equation 1.

## Results – Phonon dispersion curves in (Ce,La)Pd_2_Al_2_

The vibrations of atomic nuclei in the crystal structure are described by normal modes, which form the phonon dispersion curves in the Brilliouin zone. These modes are classified and labelled according to irreducible representations (IRs) of the group of $\mathbf{k}$ vector (little group) $\mathcal {G}_\mathbf{k}$ contained in mechanical representation. Before finding IR of $\mathcal {G}_\mathbf{k}$ it has to be noted that we have symmorphic (SSG) and non-symmorphic space groups (NSG). In the case of SSG, all translations can be factorised, and the factor group $\bar{\mathcal {G}}_\mathbf{k}$ form a point group. Unlike SSG, the NSG contain glide planes and screw axes, and consequently the $\bar{\mathcal {G}}_\mathbf{k}$ is not isomorphic to a point group in general. This means that only at the $\Gamma$ point, $\mathbf{k} =0$, the decomposition into IRs of point groups is possible for both SSG and NSG. In the following text, the phonon modes and phonon branches are labelled according to the solid state notation used in^18^. Only at the $\Gamma$ point, where the little group $\mathcal {G}_\mathbf{k}$ is identical to the point group, the notation used in chemistry is given in brackets. Away from the centre of the Brilliouin zone, the symmetry of phonon dispersion curves were determined by using the compatibility relations in^18^.

The point group of the $P\text {4}/nmm$ space group is D$_{4h}$. For symmetry determination of the phonon dispersion curves at the $\Gamma$ point, we used the direct product of equivalence representation and representation of the polar vector:

3 $$\begin{aligned} \begin{array}{l} \left( 5\text {A}_{1g} + 3\text {A}_{2u} + 2\text {B}_{2u}\right) \times \left( \text {E}_u + \text {A}_{2u} \right) = 5\text {E}_u + 5\text {E}_g + 5\text {A}_{2u} + 3\text {A}_{1g} + 2\text {B}_{1g}. \end{array} \end{aligned}$$

There are therefore 20 different phonon modes (30 with the consideration of degeneracy) at the $\Gamma$ point. This is in agreement with the fact that 10 atoms are in the primitive unit cell of CePd_2_Al_2_. All the 30 modes are listed in the Supplementary material and labelled with a number (#No.) increasing with their energy. These numbers are used further in the text.

**Figure 1:**
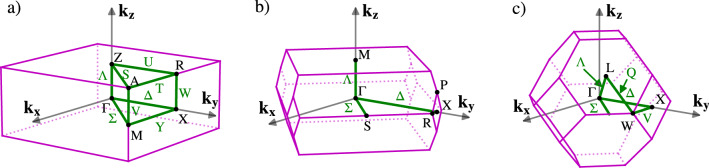
First Brilliouin zones and labels of higher symmetry points and directions, with respect to^18^. (**a**) Simple tetragonal lattice (CePd_2_Al_2_). (**b**) Body centered tetragonal lattice (CeCuAl_3_). (**c**) Reciprocal body centered cubic lattice (CeAl_2_-direct face centered cubic).

**Figure 2:**
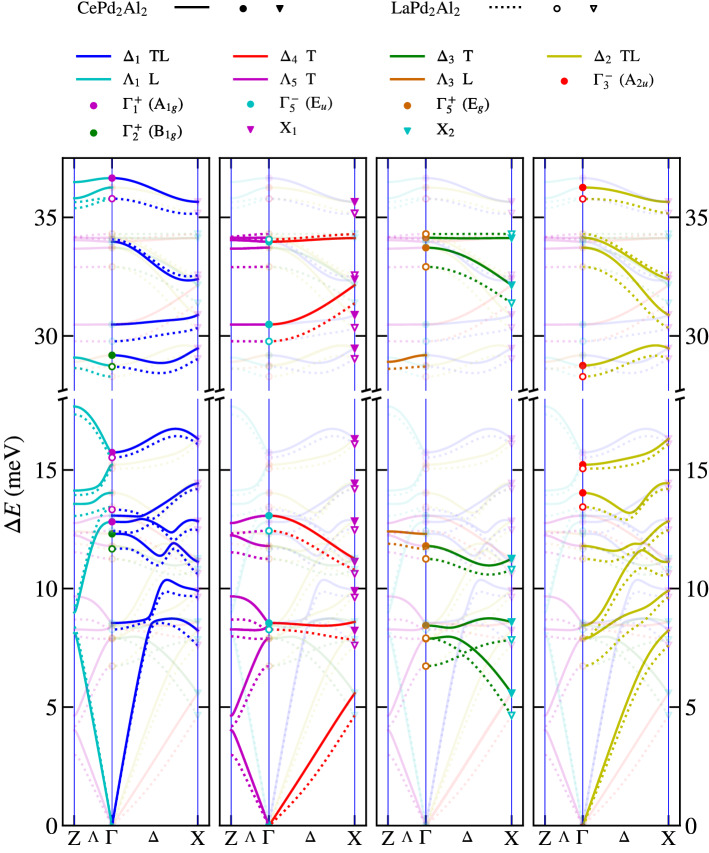
Symmetry analysis of 30 calculated phonon modes for (Ce,La)Pd_2_Al_2_, considering degeneracy. The abbreviation TL signifies the mixed character of longitudinal and transversal polarisation and T signifies a pure transversal character. Each panel highlight modes and branches with selective symmetry, the corresponding labels are above each panel. For details see chapter Results–Phonon dispersion curves in (Ce,La)Pd_2_Al_2_.

The aforementioned symmetry analysis allows us to separate the calculated phonon branches in^12^ according to their symmetry within the Brilliouin zone. Figure 1a shows higher symmetry points and directions in the primitive tetragonal unit cell using the notation in^18^. Our measurements were performed in $\Lambda$ and $\Delta$ directions. Our symmetry analysis, shown in Fig. 2, is therefore limited to these directions. The four panels highlight always one symmetry type of phonon branches and the rest of them are plot in half-transparent colour for the sake of clarity. The non-symmorphic nature (a glide plane in the basal plane) of our space group is responsible for the two fold degeneracy of modes at the X point, where always two phonon branches are stuck together and consequently their slope is not perpendicular to the Brilliouin zone boundary, see X_1_ and X_2_ phonon modes in Fig. 2. The glide plane is also responsible for the mixed character of longitudinal and transversal $\Delta _1$ and $\Delta _2$ branches. Only $\Delta _3$ and $\Delta _4$ are pure transversal with displacement in the *x*-direction. The $\Delta _1$ and $\Delta _2$ phonon branches with a mixed character often tend to cross each other, which leads to frequent anticrossings. The dispersion curves for CePd_2_Al_2_ and LaPd_2_Al_2_ are very similar. Most of them appear at higher energies for CePd_2_Al_2_ compared to LaPd_2_Al_2_. This can be understood if we consider that the CePd_2_Al_2_ unit cell has a smaller volume. The detailed discussion about differences in these two homologs together with partial density of states is presented in^12^.

**Figure 3:**
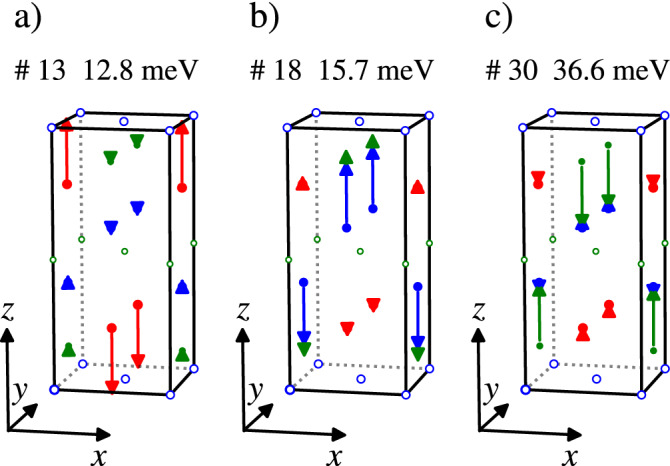
The atomic displacement of atoms for the $\Gamma _1^+$ (A$_{1g}$) phonon mode symmetry. Calculated at^12^ for CePd_2_Al_2_. For clarity the displacement of each atom is 4 times enlarged. The labels #13 #18 #30 correspond to the notation used in Supplementary information, which labels the modes for a given $\mathbf{q}$ with increasing energy. The red, blue and green colours correspond to Ce, Pd and Al ions, respectively. The open circles represents static atoms in the mode.

**Figure 4:**
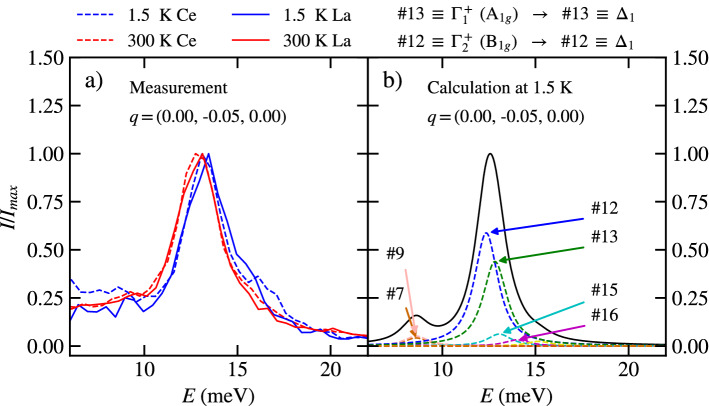
(**a**) The measured inelastic spectra at the 0 1 8 Brillouin zone ($\mathbf{q}=(0.00, -0.05, 0.00)$) temperatures 1.5 and 300 K. The results are shown for CePd_2_Al_2_ and LaPd_2_Al_2_ compounds. (**b**) The calculated intensity (full black line) is the sum of all 30 modes at the 0 1 8 Brillouin zone for CePd_2_Al_2_, the most intense modes are labelled.

The aim of our experimental study is a mapping of the selected phonon dispersion curves and especially modes with the A$_{1g}$ symmetry as this symmetry was assumed to be involved in the magneto-elastic coupling^5^. The A$_{1g}$ phonon modes are present only at the $\Gamma$ point, where we find three of them. Figure 3 shows the displacement amplitude of atoms in the unit cell for all three A$_{1g}$ modes. The energy of the phonon mode, involved in the magneto-elastic coupling, should be comparable with the energy of magnetic excitation from ground state in the unperturbed level scheme based on standard CEF theory. Based on the magnetic excitations observed by inelastic neutron scattering^5^, this restricts our attention to the A$_{1g}$ mode at 12.8 meV with a dominant motion of Ce ions, see Fig. 3. Direct measurement of inelastic x-ray scattering at the $\Gamma$ point is not possible, because of the strong elastic signal, therefore the measurements were performed in the close vicinity of the $\Gamma$ point at $\mathbf{q} = (0.00, -0.05, 0.00)$. The 0 1 8 Brilliouin zone was selected for measurements as the calculated intensity of A$_{1g}$ mode at 12.8 meV is dominant in the spectra in this zone. Figure 4a shows the temperature dependence of the $\Delta _1$ phonon mode (#13) related by compatibility relations with the A$_{1g}$ mode at 12.8 meV at the $\Gamma$ point. The results are compared with calculated spectra in 4b. The measured spectra at the 0 1 8 Brillouin zone are almost identical for both CePd_2_Al_2_ and LaPd_2_Al_2_. The temperature dependence of the phonon mode energy is negligible with only a slight shift to higher energy at low temperature. The spectral width of modes is similar at room and lowest temperature and also for CePd_2_Al_2_ and LaPd_2_Al_2_ homologs.

**Figure 5:**
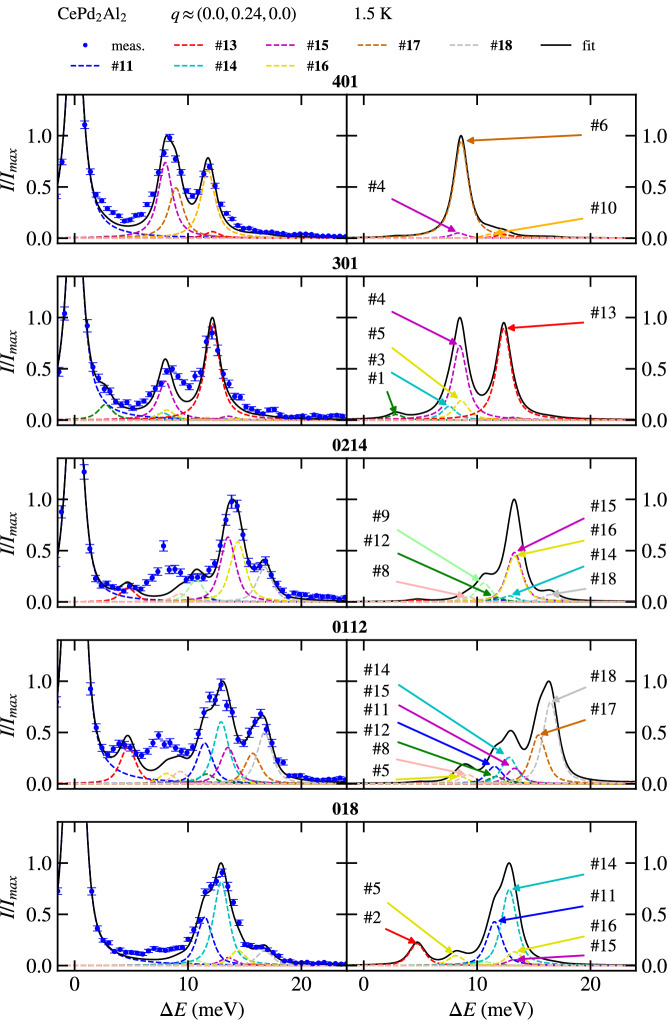
The fit of measured data at $\mathbf{q} = (0.00, 0.24, 0.00)$ on the left and calculated intensity based on dynamical structure factor on the right. Each pair of graphs correspond to the given Brillouin zone specified in bold above them. The most intense modes are labelled. The colour of the selected mode is the same across the Brillouin zones. The legend above shows modes of which energies were possible to determine conclusively.

The other phonon modes need also a closer inspections, since the magneto-elastic coupling in CeAl_2_, which is the archetypal example, affects not only the given phonon mode, but the whole corresponding branches. From this point of view, it is important to study the $\mathbf{q}$ dependencies of phonon branches and compare them with the calculated ones and with non-magnetic analogues. It is almost impossible to find such a Brilliouin zone, where only the intensity of one phonon branch will be intense and well separated from the others. This problem is partly solved by measuring at several Brilliouin zones at the same $\mathbf{q}$ position. The energy of the phonon mode is then fitted and kept the same in all Brillouin zones. The spectral line shape is given by a pseudo-Voigt function, which parameters were determined by a fit of the elastic line. This procedure improves a lot the data analysis, but still the intensity ratio between modes based on a dynamical structure factor has to be fixed to increase the stability of the fit. The example of such procedure is shown in Fig. 5 for CePd_2_Al_2_ compound, $\mathbf{q} \approx (0.00, 0.24, 0.00)$ at 1.5 K. It has to be noted that the measured intensity is integrated intensity from a part of reciprocal space and not an intensity at given point in reciprocal space due to the finite resolution. This could have significant influence on the measured intensity, especially where the intensity of the mode changes rapidly within the Brilliouin zone. This could also be the reason for enhanced intensity around 8 meV in the spectra, see Fig. 5. The same feature was observed for both Ce and La homologs (see comparison of measured data in Supplementary material) and cannot be therefore related to the magneto-elastic coupling.

**Figure 6:**
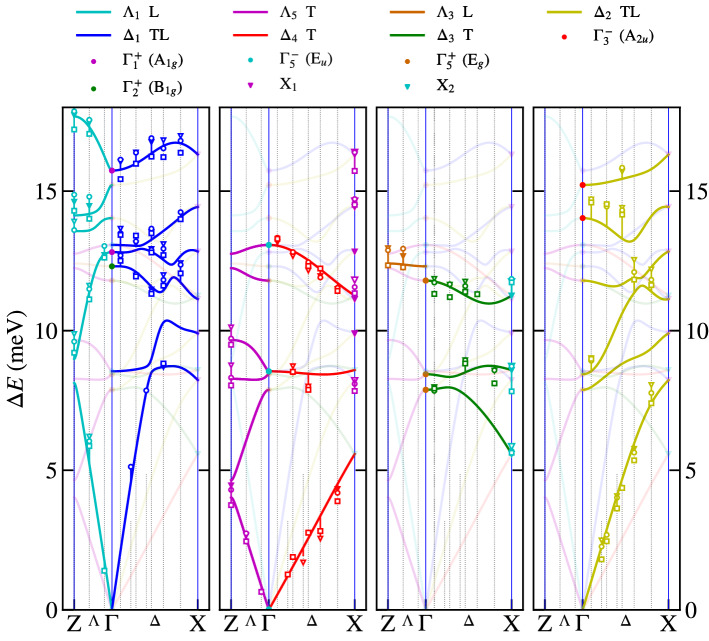
The comparison of theoretical calculation with the fit of measured inelastic X-ray scattering spectra for CePd_2_Al_2_ in energy range 0 - 17.5 meV. The $\square$, ○, $\triangledown$ symbols correspond to 1.5, 7.5, 280 K, respectively. For details of the fit, see chapter Results – Phonon dispersion curves in (Ce,La)Pd_2_Al_2_.

**Figure 7:**
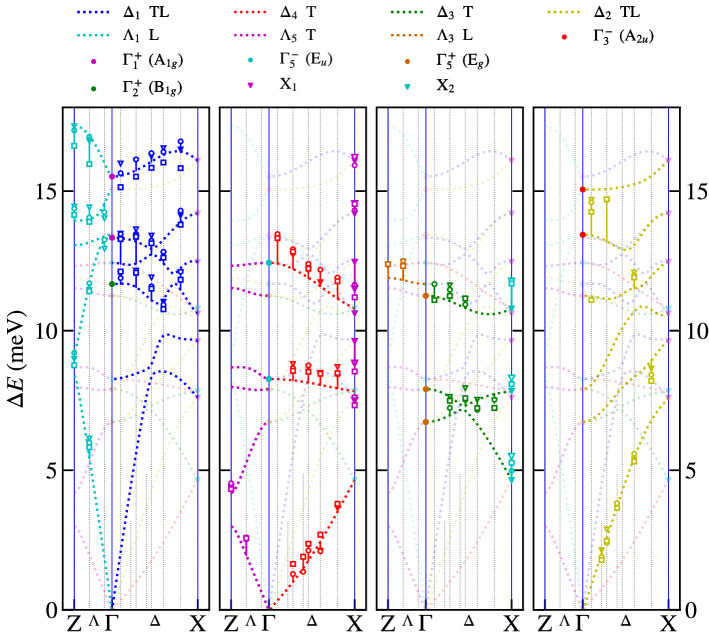
The comparison of theoretical calculation with the fit of measured inelastic X-ray scattering spectra for LaPd_2_Al_2_ in energy range 0 - 17.5 meV. The $\square$, ○, $\triangledown$ symbols correspond to 1.5, 80, 300 K, respectively. For details of the fit, see chapter Results - Phonon dispersion curves in (Ce,La)Pd_2_Al_2_.

The measurements were performed at three different temperatures 1.5, 7, and 280 K for CePd_2_Al_2_ and 1.5, 80 and 300 K for LaPd_2_Al_2_. The temperatures 7 and 80 K were selected to be close to the structural phase transition, but still above them. The results of the above mentioned analysis are shown in Figs. 6 and 7 for both Ce and La homologs, respectively. The **q**-dependences of phonon modes agree very well with the calculated ones. The temperature dependence of phonon energies is negligible, except the $\Delta _1$ and $\Lambda _1$ branches around 15.7 meV, where the motion of Pd atoms is dominant. The significant difference between calculated and measured data is around 12.4 meV $\Delta _4$ for LaPd_2_Al_2_, in contrary the results agree very well for CePd_2_Al_2_ in this symmetry. The raw measured data of Ce and La homologs are almost identical (see Supplementary material), which suggest better agreement between measurement and calculation for CePd_2_Al_2_. A significant shift of the $\Delta _2$ branch to the higher energy of around 14.0 meV was observed for both compounds, although better agreement is found again for CePd_2_Al_2_.

## Discussion – quasi-bound state

The phonon dispersion study in (Ce,La)Pd_2_Al_2_ is motivated by the proposed formation of a quasi-bound state in this compound. Therefore we discuss the experimental results with respect to the theoretical model describing this quantum state.

The CEF magnetic excitations are usually non-dispersive within Brillouin zone, unlike phonons. The strength of any magneto elastic coupling, $g_0^{\mu }$, depends on $\varvec{k}$ and on a given mode *s*. The most important contribution to the interaction term can be expressed as in^8^:

4 $$\begin{aligned} \begin{array}{l} {\hat{H}}_{int} = - \sum \limits _{l \tau } \sum \limits _{\varvec{k} s \mu } g_0^{\mu }\left( \varvec{k} s, \tau \right) \left( {\hat{a}}_{\varvec{k}s} + {\hat{a}}_{\varvec{-k}s}^{+} \right) {\hat{O}}_{\mu }\left( \varvec{R}_{l \tau } \right) e^{-\mathrm {i}\varvec{k} \cdot \varvec{R}_{l \tau }}, \end{array} \end{aligned}$$

where $l, \tau$ indexes run over primitive unit cell and atoms in the basis, respectively. The ${\hat{a}}$ and ${\hat{a}}^{+}$ are the annihilation and creation operators for phonons. The operator ${\hat{O}}_{\mu }$ represents CEF states and the ${\hat{O}}_{\mu } e^{-\mathrm {i}\varvec{k} \cdot \varvec{R}_{l \tau }}$ should transform as the phonon mode $\varvec{k}s$ (as the IR of little group). The ${\hat{O}}_{\mu }$ itself then transforms like IRs of a factor group of the little group. The factor group is a point group in case of SSG, while it is not generally isomorphic to a point group in case of NSG, see chapter Results - Phonon dispersion curves in (Ce,La)Pd_2_Al_2_. This has a consequence that the direct finding of ${\hat{O}}_{\mu }$ operators for some high symmetry points in the Brillouin zone is not possible in NSG. The situation is much easier at the $\Gamma$ point ($\varvec{k} = 0$), where the little group is a point group for SSG and also NSG. This simplification is used in the non-dispersive model for the interaction term^8^:

5 $$\begin{aligned} \begin{array}{l} {\hat{H}}_{int} = - \sum \limits _{\mu } g_{\mu } \left( {\hat{a}}_{\mu } + {\hat{a}}_{\mu }^{+} \right) {\hat{O}}_{\mu }, \end{array} \end{aligned}$$

where ${\hat{a}}_{\mu }^{+}$ and ${\hat{a}}_{\mu }$ are creation and annihilation operators for phonons $\mu$, $g_{\mu }$ is a magnetoelastic coupling parameter. This interaction term was used for theoretical description in CeAl_2_^8^, CeCuAl_3_^6^, CeAuAl_3_^7^ and CePd_2_Al_2_^5^.

Determination of the magneto-elastic coupling for various phonon modes and the decision about the dominant contribution is a highly non-trivial task. In the case of cubic CeAl_2_ (the first Brillouin zone is shown in Fig. 1c this was assisted by experimental studies of phonon dispersion curves. Softening and broadening of the $\Gamma _{25}^{'}$ phonon mode was observed there, suggesting this mode to be considered in the model^10^. Such a direct observation of a quasi-bound state in the phonon dispersions of CePd_2_Al_2_, CeCuAl_3_ and CeAuAl_3_ has not been reported, yet.

Before we turn to our results on CePd_2_Al_2_, lets briefly discuss the case of CeCuAl_3_ and CeAuAl_3_. The previous single crystal studies of the phonon spectra in these two compounds were not focused on specific phonon modes with symmetry considered in the theoretical model. The crystal structure of CeAuAl_3_ was determined to be the BaNiSn_3_ structural type^19^ with *I*4 mm space group. The space group is symmorphic and the point group is C$_{4v}$. The interaction term of model Hamiltonian uses the ${\hat{O}}_2^2 = {\hat{J}}_x^2 - {\hat{J}}_y^2$ operator^7^. This means that the corresponding phonon modes should transform as $x^2-y^2$ basis function, which corresponds to the $\Gamma _3$ (B_1_) phonon mode. Away from the $\Gamma$ point, using the dispersive model, such symmetry could be found in V, M points and $\Lambda$ direction, because the corresponding factor groups are isomorphic to the C$_{4v}$ point group, see Fig. 1b. The crystal structure of CeCuAl_3_ is often reported the same as that of CeAuAl_3_, so the conclusions based on symmetry analysis would be valid also here. However the crystal structure of CeCuAl_3_ seems to be more complex than CeAuAl_3_. The partial site mixing on Cu and Al Wyckoff position was previously observed^19–21^. The model of a quasi-bound state is based on the fact, that Ce ions in the crystal structure have identical environments, which is not expected for the solid solution compounds. Also the study of magnetic excitations in the CeCu$_x$Al$_{4-x}$ system by inelastic neutron scattering^22^ shows the continuous evolution of more than three magnetic excitations in the spectra, which is the sign of non-identical Ce environments.

In the case of CePd_2_Al_2_ the relevant phonon modes were suggested to be the A$_{1g}$ modes^5^. We note that the A$_{1g}$ IR transforms as $x^2+y^2,z^2$ basis functions in the studied symmetry D$_{4h}$. Only spherical harmonics Y$_{0,0}$ and Y$_{2,0}$ for $l<9$ transform as A$_{1g}$, therefore ${\hat{O}}_{\mu }$ could be considered as ${\hat{O}}_2^0 = 3 {\hat{J}}_z^2 - J(J+1)$ for CePd_2_Al_2_. The situation for reciprocal space points away from the $\Gamma$ point is more complicated, than in CeAuAl_3_, because the *P*4/nmm is NSG. The factor group is isomorphic with the point groups only at $\Gamma$, Z, V, W points and in the $\Lambda$ direction. The A$_{1g}$ representation is present only at Z (point group D$_{4h}$).

Based on these arguments, our experimental study was focused on the A$_{1g}$ modes and on modes in the $\Delta$ and $\Lambda$ directions. There are three A$_{1g}$ modes at $\Gamma$ point at 12.8, 15.7 and 36.6 meV. Taking into account that the energy of the mode should be comparable to the energy of CEF excitation from the ground state, the mode at 36.6 meV can be excluded. The measurement of related phonon branches to A$_{1g}$ modes at 12.8, 15.7 meV in the vicinity of the $\Gamma$ point doesn’t show any broadening or softening. The other mode symmetries at the $\Gamma$ point (E$_g$, E$_u$, A$_{2u}$, B$_{1g}$ modes) in the energy interval 7.5–17 meV show a similar temperature dependence as the A$_{1g}$ modes, or their energy is constant in temperature. Looking at the phonon dispersion curves in the $\Delta$ and $\Lambda$ directions, we didn’t observed any softening, which also says that in the X and Z points the temperature behaviour is similar. Comparison of raw measured spectra in Supplementary materials of CePd_2_Al_2_ and LaPd_2_Al_2_ homologues show no significant difference, which could be ascribed to the expected quasi-bound state.

The crystal lattice of CePd_2_Al_2_ and LaPd_2_Al_2_ is distorted below 6 K and 60 K, respectively. At 1.5 K, the larger distortion is found for LaPd_2_Al_2_. Therefore we will discuss the influence of distortion on phonon dispersion curves in LaPd_2_Al_2_ homologue. During the distortion we lost the four-fold axis, just two-fold axis remains. This lowering of symmetry leads to decrease of dimension of IRs in mechanical representation. It leads to the splitting of two times degenerated phonon modes at the $\Gamma$ point ($\Gamma _5^{-}$(E$_u$), $\Gamma _5^{+}$(E$_g$)) and also in the $\Lambda$ direction ($\Lambda _5$). On the other hand in the $\Delta$ direction the distortion leads only to the modification of their $\mathbf{q}$ dependencies. In the X point the two fold-degeneracy of phonon modes is kept, because it requires the NSG origin as in the tetragonal case. Fortunately the effect of the distortion is small and the difference is very often smaller than in comparison of Ce and La homologues (see Supplementary information). Therefore we compare the measurement at low temperature with phonon dispersion curves at room temperature in the Figs. 6 and 7 .

We can conclude that our measurements show very good agreement of the observed phonon dispersion curves with the calculated ones without introducing the quasi-bound state. The previous idea^5^ that the A$_{1g}$ phonon modes may be the strongest coupled to the magnetic excitations can be excluded by our study. Other mode symmetries at the $\Gamma$ point also seem to be unaffected by the quasi-bound state, which opens the question of the meaning of application of a dispersion-less model and even about the presence of a quasi-bound state in CePd_2_Al_2_. Beside CePd_2_Al_2_ we explored the mode symmetries and identified the high symmetry points in the first Brillouin zone of CeAuAl_3_ and CeCuAl_3_, at which the following phonon study should be focused in order to follow the consequence of a non-dispersive model of the possible quasi bound state.

## Supplementary information

Supplementary Information.

## Data Availability

Raw data were generated at the SPring-8 large-scale facility. Derived data supporting the findings of this study are available within the article and its Supplementary information for: Lattice dynamics in CePd_2_Al_2_ and LaPd_2_Al_2_.

## References

[1] Ruminy M (2016). Crystal-field parameters of the rare-earth pyrochlores R_2_Ti_2_O_7_ (*R* = Tb, Dy, and Ho). Phys. Rev. B.

[2] Gaudet J (2018). Magnetoelastically induced vibronic bound state in the spin-ice pyrochlore Ho_2_Ti_2_O_7_. Phys. Rev. B.

[3] Loewenhaupt M, Steglich F (1977). The magnetic behaviour of CeAl_2_ studied by neutron scattering. Physica B.

[4] Mühle E, Goremychkin EA, Natkaniec I (1989). Inelastic Neutron Scattering on (Pr, La)Ni_2_ and (Pr, Y)Ni_2_. J. Mag. Mag. Mater..

[5] Chapon LC, Goremychkin EA, Osborn R, Rainford BD, Short S (2006). Magnetic and structural instabilities in CePd_2_Al_2_ and LaPd_2_Al_2_. Physica B.

[6] Adroja, D. T. *et al.* Vibron Quasibound State in the Noncentrosymmetric Tetragonal Heavy-Fermion Compound CeCuAl_3_. *Phys. Rev. Lett.* **108**, 216402–1 – 5, 10.1103/PhysRevLett.108.216402 (2012). 10.1103/PhysRevLett.108.216402 23003286

[7] Čermák P (2019). Magnetoelastic hybrid excitations in CeAuAl_3_. Proc. Natl. Acad. Sci. USA.

[8] Thalmeier P (1984). Theory of the bound state between phonons and a CEF excitation in CeAl_2_. J. Phys. C Solid State Phys..

[9] Godet M, Walker E, Purwins H-G (1973). Preparation of single crystals of CeAl_2_ by the Czochralski method. J. Less-Common Metals.

[10] Reichardt W, Nücker N (1984). Phonon softening in CeAl_2_. J. Phys. F Met. Phys..

[11] Tsutsui S, Kaneko K, Pospíšil J, Haga Y (2018). Inelastic X-ray scattering of RTAl_3_ (*R* = La, Ce, *T* = Cu, Au). Physica B.

[12] Legut D, Diviš M, Doležal P, Zhang SH, Javorský P (2020). Ab initio calculations of the crystal field and phonon dispersions in CePd_2_Al_2_ and LaPd_2_Al_2_. J. Phys. Condensed Matter.

[13] Doležal P (2017). Czochralski growth of LaPd_2_Al_2_ single crystals. J. Crystal Growth.

[14] Doležal P (2019). Structural instability in CePd_2_(Al, Ga)_2_ and LaPd_2_(Al, Ga)_2_. J. Alloys Compd..

[15] Baron AQR (2000). An X-ray scattering beamline for studying dynamics. J. Phys. Chem. Solids.

[16] Baron, A. Q. R. Phonons in crystals using inelastic X-ray scattering. *J. Spectrosc. Soc. Japan* **58**, 205–2014, arXiv:0910.5764 - english version (2009).

[17] Als-Nielsen, J. & McMorrow, D. *Elements of Modern X-ray Physics* (Wiley, 2001).

[18] Miller SC, Love WF (1967). Tables of Irreducible Representations of Space Groups and Co-Representations of Magnetic Space Groups.

[19] Franz C (2016). Single crystal growth of Ce*T*Al_3_ (T = Cu, Ag, Au, Pd and Pt). J. Alloys Comp..

[20] Chlan, V. *et al.* Local atomic arrangement in LaCuAl_3_ and LaAuAl_3_ by NMR and density functional theory. *J. Phys. Condens. Matter* **31**, 385601, 10.1088/1361-648X/ab27ac (2019). 10.1088/1361-648X/ab27ac 31170703

[21] Matsumura M, Kawamura Y, Yoshina M, Nishioka T, Kato H (2009). ^27^Al-NQR study in BaNiSn_3_-type CeCuAl_3_. J. Phys. Conf. Ser..

[22] Klicpera M (2017). Magnetic structure and excitations in CeCu_*x*_Al_*4-x*_ system. Inorganic Chem..

