# Supplementary material for: Lattice dynamics in CePd2Al2 and LaPd2Al2
Source: Sci Rep. 2021 Oct 22;11:20878. doi: 10.1038/s41598-021-99904-7 (PMC8536711; doi:10.1038/s41598-021-99904-7)
Supplement: Supplementary file 1 — Supplementary Information. [file 41598_2021_99904_MOESM1_ESM.pdf]

Supplementary information for:

Lattice dynamics in $CePd_2Al_2$ and $LaPd_2Al_2$

P. Doležal[1], P. Cejpek[1], S. Tsutsui[2,3], K. Kaneko[4], D. Legut[5], K. Carva[1], and P. Javorský[1]

[1] *Charles University, Faculty of Mathematics and Physics, Department of Condensed Matter Physics, Ke Karlovu 5, 121 16 Prague 2, Czech Republic*

[2] *Japan Synchrotron Radiation Research Institute (JASRI), SPring-8, Sayo, Hyogo 679-5198, Japan*

[3] *Institute of Quantum Beam Science, Graduate School of Science and Engineering, Ibaraki University, Hitachi, Ibaraki 316-8511, Japan*

[4] *Materials Science Research Center, Japan Atomic Energy Agency, Tokai, Ibaraki 319-1195, Japan*

[5] *IT4Innovations, VSB-Technical University of Ostrava, 17. listopadu 2172/15, 708 00 Ostrava, Czech Republic*

Structural transition in (Ce,La)Pd_2Al_2

A low-temperature X-ray diffraction study of (Ce,La)Pd_2Al_2 compounds was performed on a refurbished Siemens D500 $\theta - \theta$ diffractometer, using Cu-K$\alpha_{1,2}$ radiation. The intensity was counted by a Mythen 1K position sensitive detector. The samples were placed in He atmosphere within a cryostat, to maintain a qood thermal equilibrium. The powder sample was placed on a sapphire cube connected to the cold finger of the He closed cycle, which was used for cooling down to 3 K. The reciprocal space map of selected diffraction was measured in the same setup, but the piezoelectric rotator was used for sample alignment instead of the sapphire cube.

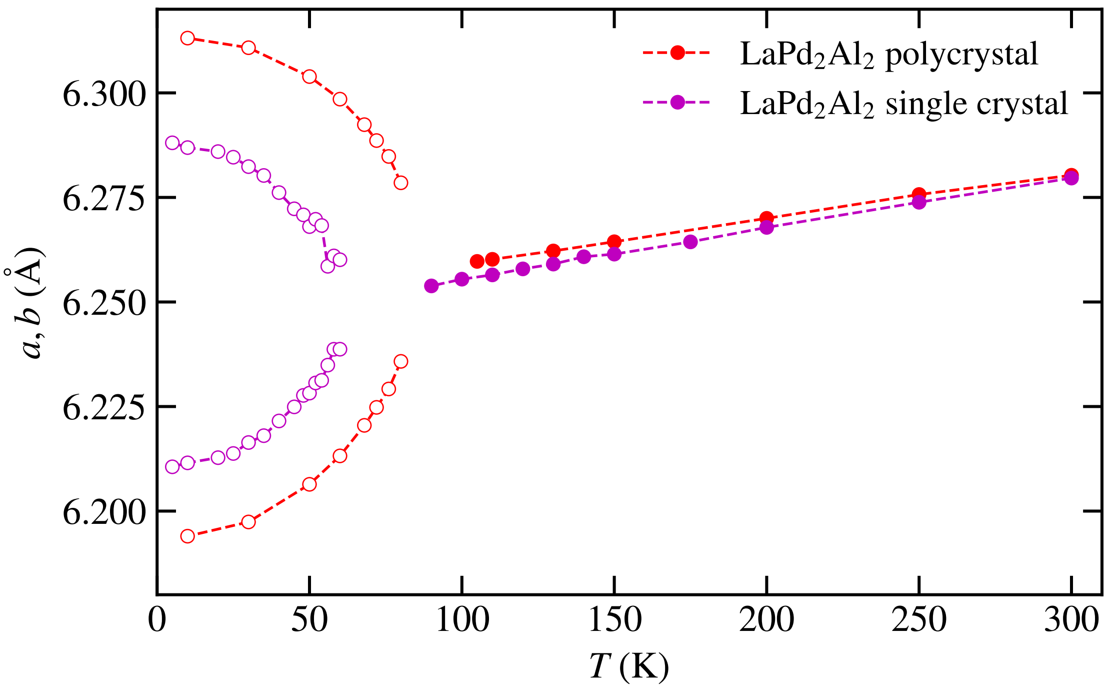

Figure 1: Comparison of magnitudes and transition temperatures of the structural distortion in $LaPd_2Al_2$. The lattice parameters of the polycrystalline sample were determined by Rietveld refinement of X-ray powder pattern. The single crystal lattice parameters were calculated from temperature dependence of the $(420)_T$ diffraction of a single crystalline sample. The lattice parameters are given with respect to the orthorhombic unit cell.

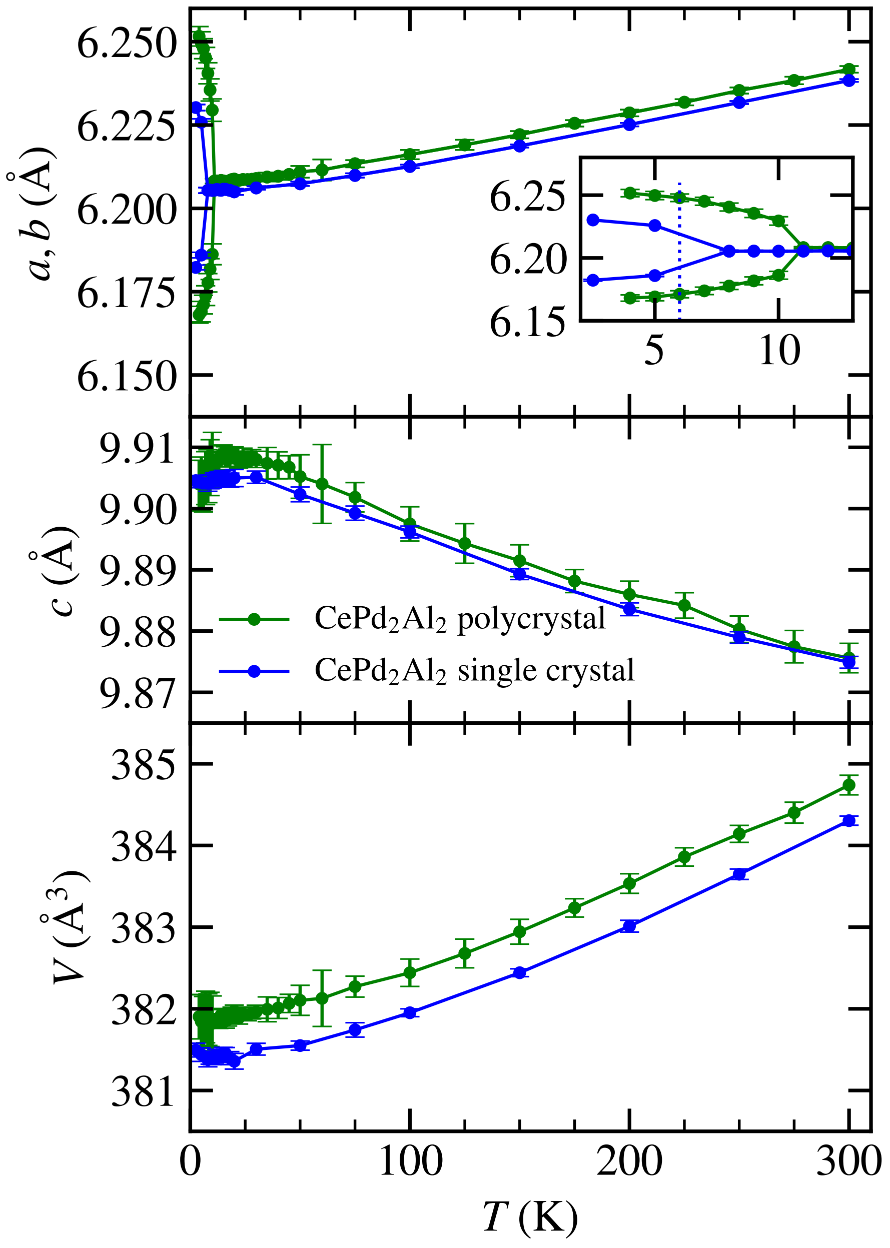

Figure 2: Results of Rietveld refinement of X-ray powder pattern for poly and single crystalline $CePd_2Al_2$ sample. The inset shows basal plane lattice parameters around the transition temperatures.

Phonon modes in $CePd_2Al_2$ at Γ point

The atomic displacement in the phonon modes, shown at Fig. 3 and Fig. 4, are results of calculation using [1]

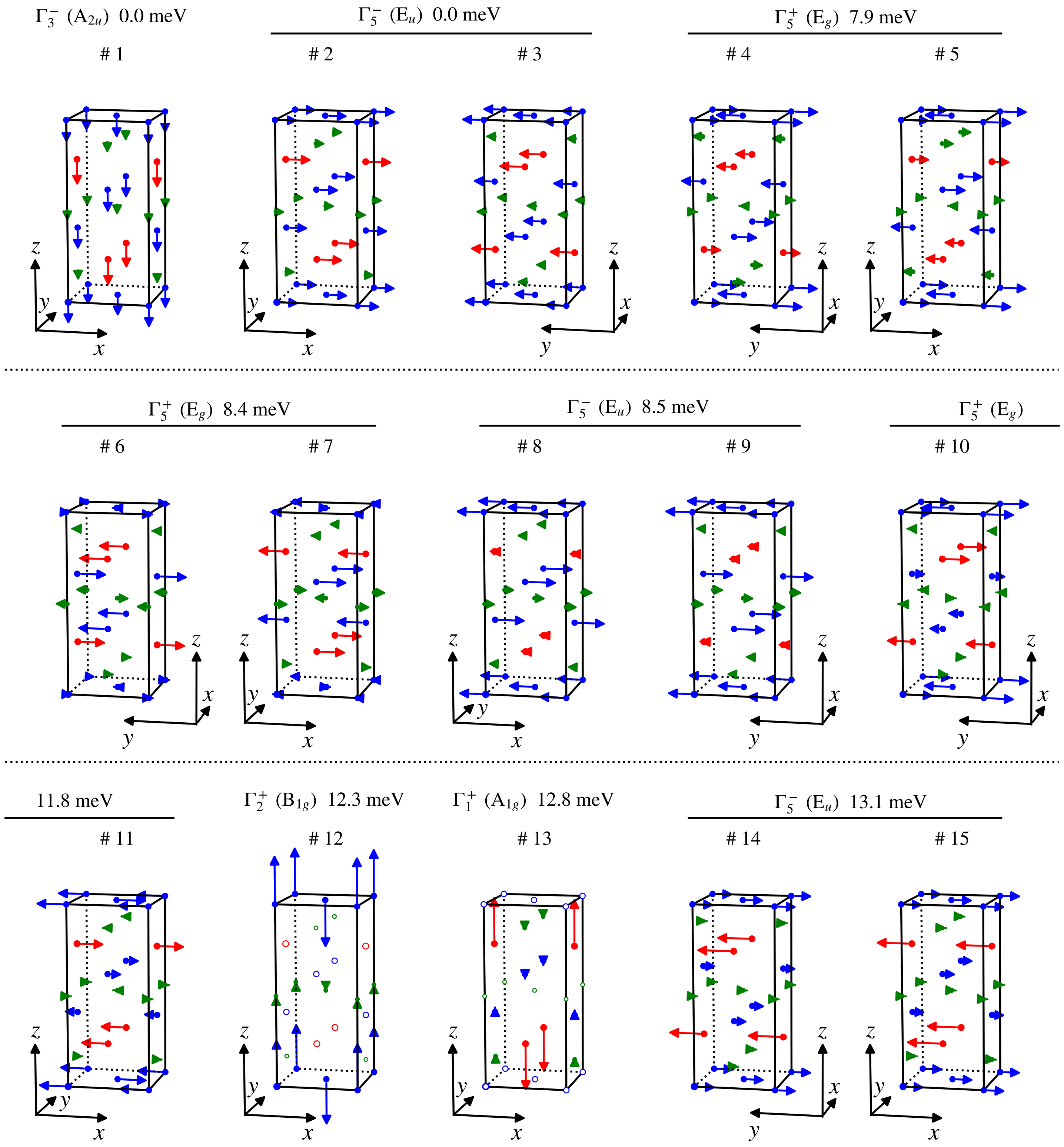

Figure 3: Displacement of atoms in phonon modes at the Γ point. The red, blue and green colour stands for Ce, Pd and Al ions, respectively. The atomic displacement is 4 times enlarged for clarity. The black solid line above the unit cells marks the 2D phonon modes and groups together the x and y-polarisation. The rest (#16 - #30) phonon modes are shown in Fig. 4.

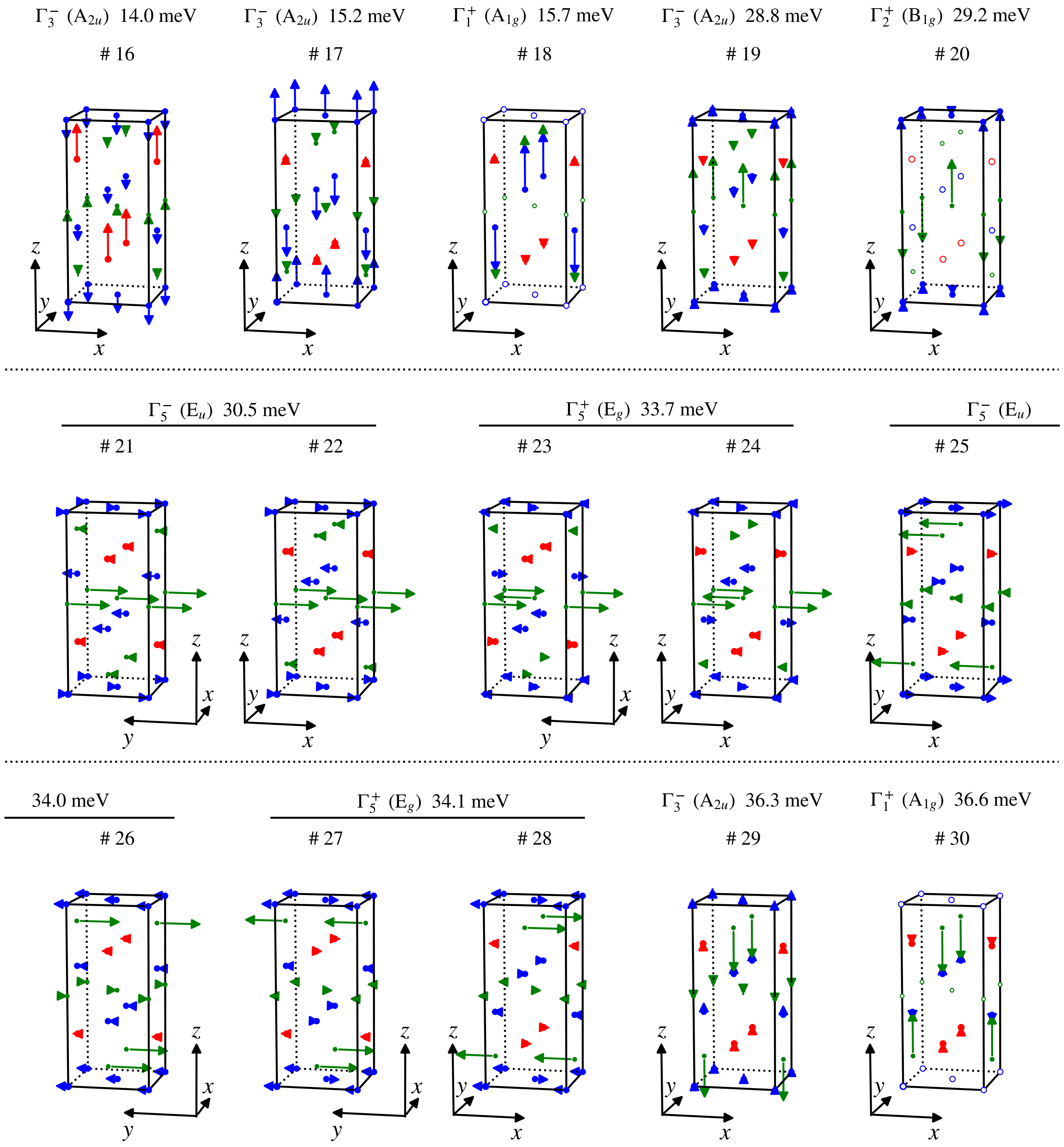

Figure 4: Atomic displacement of atom in phonon modes at Γ point (#16 - #30). Continuation of Fig. 3.

Phonon dispersion curves in distorted $LaPd_2Al_2$

The influence of structural distortion on phonon dispersion curves is shown in Fig. 5. The measurements were performed at Δ and Λ directions. Only one dimension irreducible representations and consequently non-degenerated phonon branches can be found in the Δ direction. The distortion therefore only slightly shifts and modifies their dependence. The situation is different at Γ and in Λ direction, where the two-fold degenerated phonon modes (Λ_5, $\Gamma_5^-(\mathrm{E}_u)$, $\Gamma_5^+(\mathrm{E}_g)$) are split in orthorhombic symmetry. The colours of phonon branches are kept the same before and after transition for better comparison in Fig. 5, but we have to keep in mind, that symmetry in orthorhombic structure is different. On the other hand the distorted lattice is still very close to the tetragonal one and it leads only to small differences, see Fig. 5.

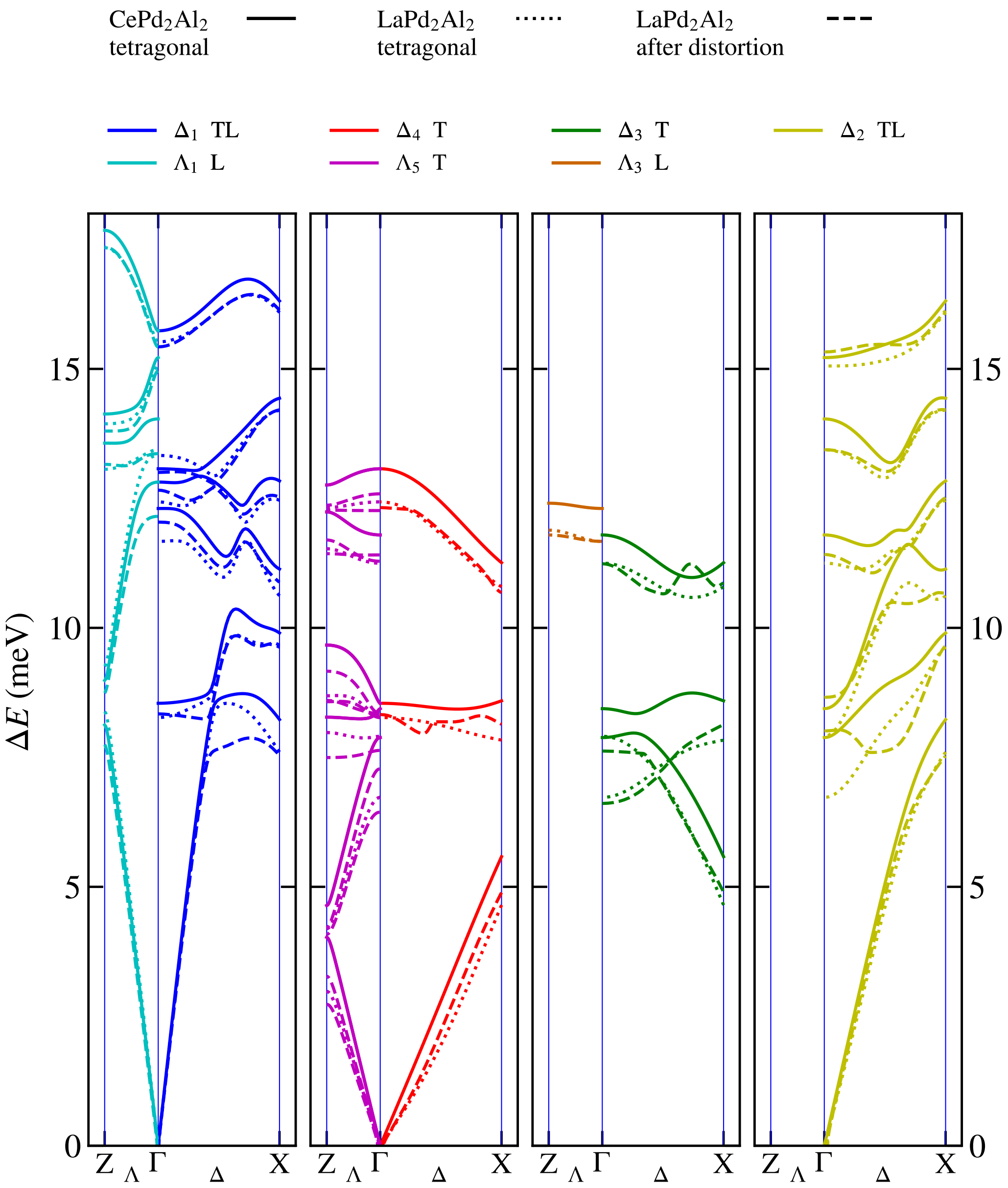

Figure 5: Comparison of phonon dispersion curves in tetragonal $CePd_2Al_2$ and $LaPd_2Al_2$ with the phonon dispersion curves in distorted $LaPd_2Al_2$ compound. The colours represents the symmetry of phonon branches at tetragonal lattice. The influence of structural distortion is described above in the text.

Inelastic X-ray spectra in (Ce,La)Pd_2Al_2

Figures 6 - 10 show the measured inelastic spectra at different Brillouin zones. The measured curves are normalised to unity at the maximum of the inelastic spectra, which allows the direct comparison between 300 K and 1.5 K and mainly the comparison between Ce and La homologs.

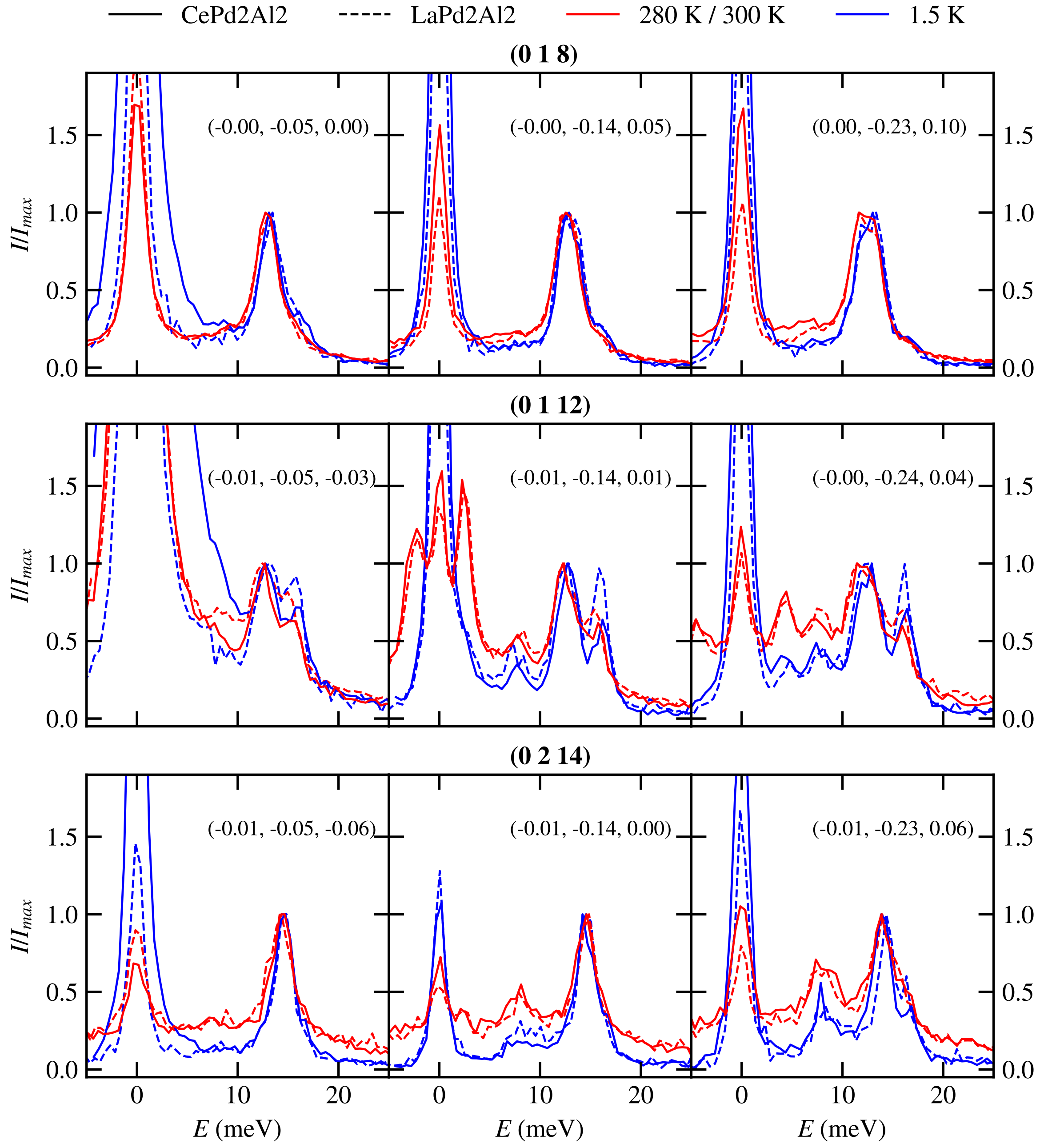

Figure 6: Comparison of measured inelastic X-ray spectra for $CePd_2Al_2$ and $LaPd_2Al_2$ at different Brillouin zones (in bold above the graphs) in Δ direction - part 1. The red colour represents temperature 280 K for $CePd_2Al_2$ and 300 K for $LaPd_2Al_2$. The position at Brillouin zone is given by the $\mathbf{q}$ vector with coordinates in brackets.

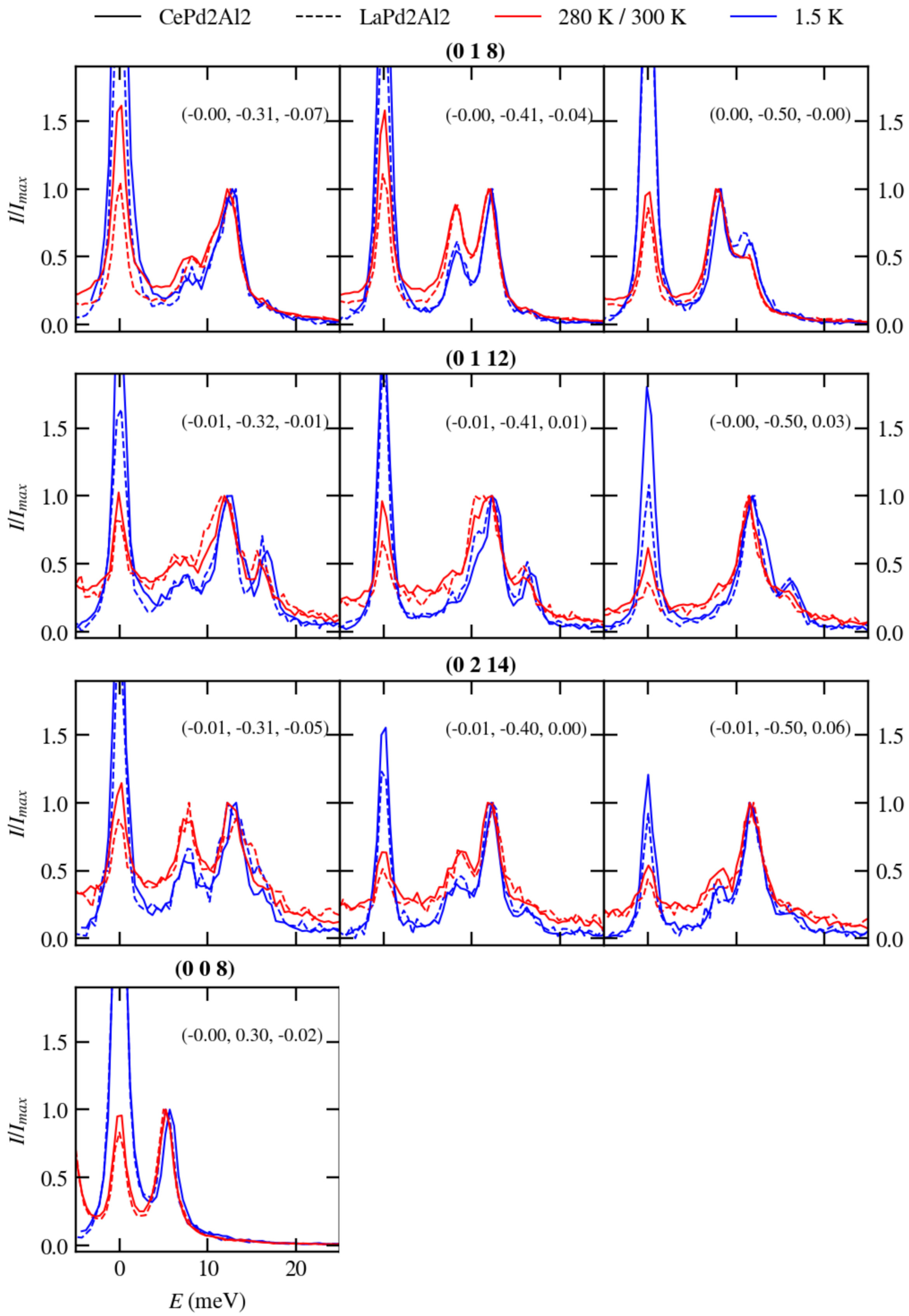

Figure 7: Comparison of measured inelastic X-ray spectra for $CePd_2Al_2$ and $LaPd_2Al_2$ at different Brillouin zones (in bold above the graphs) in Δ direction - part 2. For details, see caption of Fig.6.

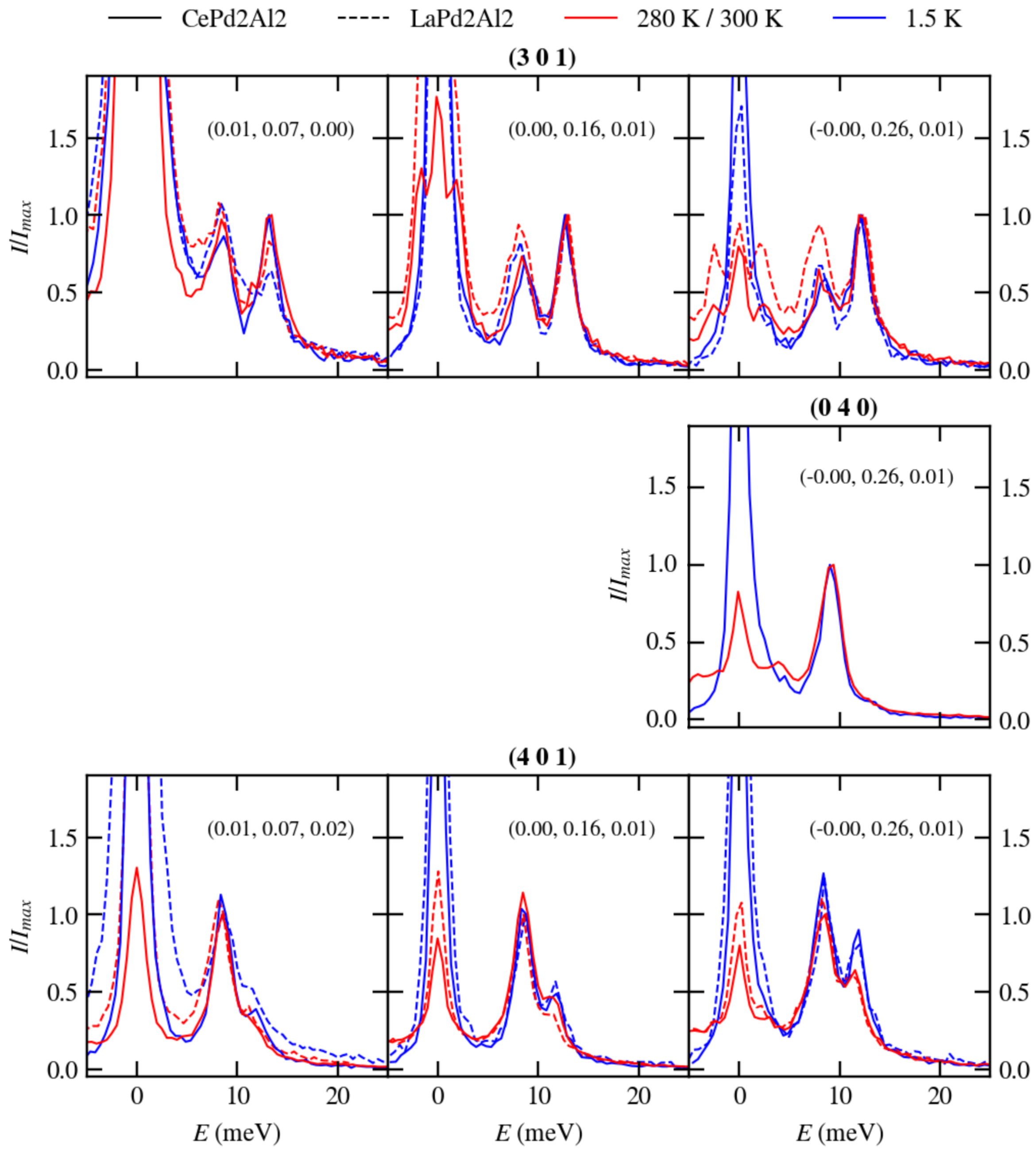

Figure 8: Comparison of measured inelastic X-ray spectra for $CePd_2Al_2$ and $LaPd_2Al_2$ at different Brillouin zones (in bold above the graphs) in the Δ direction - part 3. For details, see caption of Fig.6.

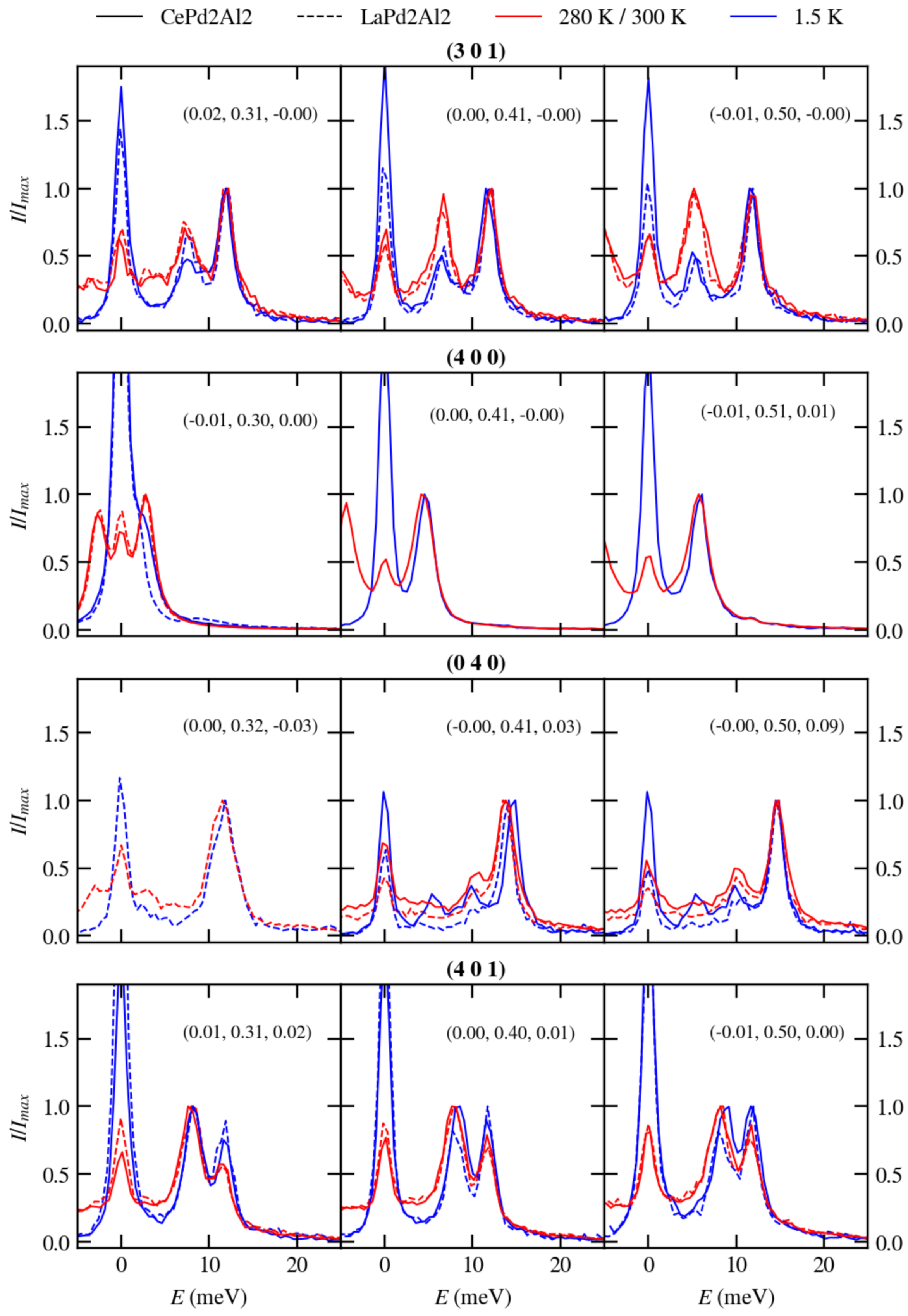

Figure 9: Comparison of measured inelastic X-ray spectra for $CePd_2Al_2$ and $LaPd_2Al_2$ at different Brillouin zones (in bold above the graphs) in the Δ direction - part 4. For details, see caption of Fig.6.

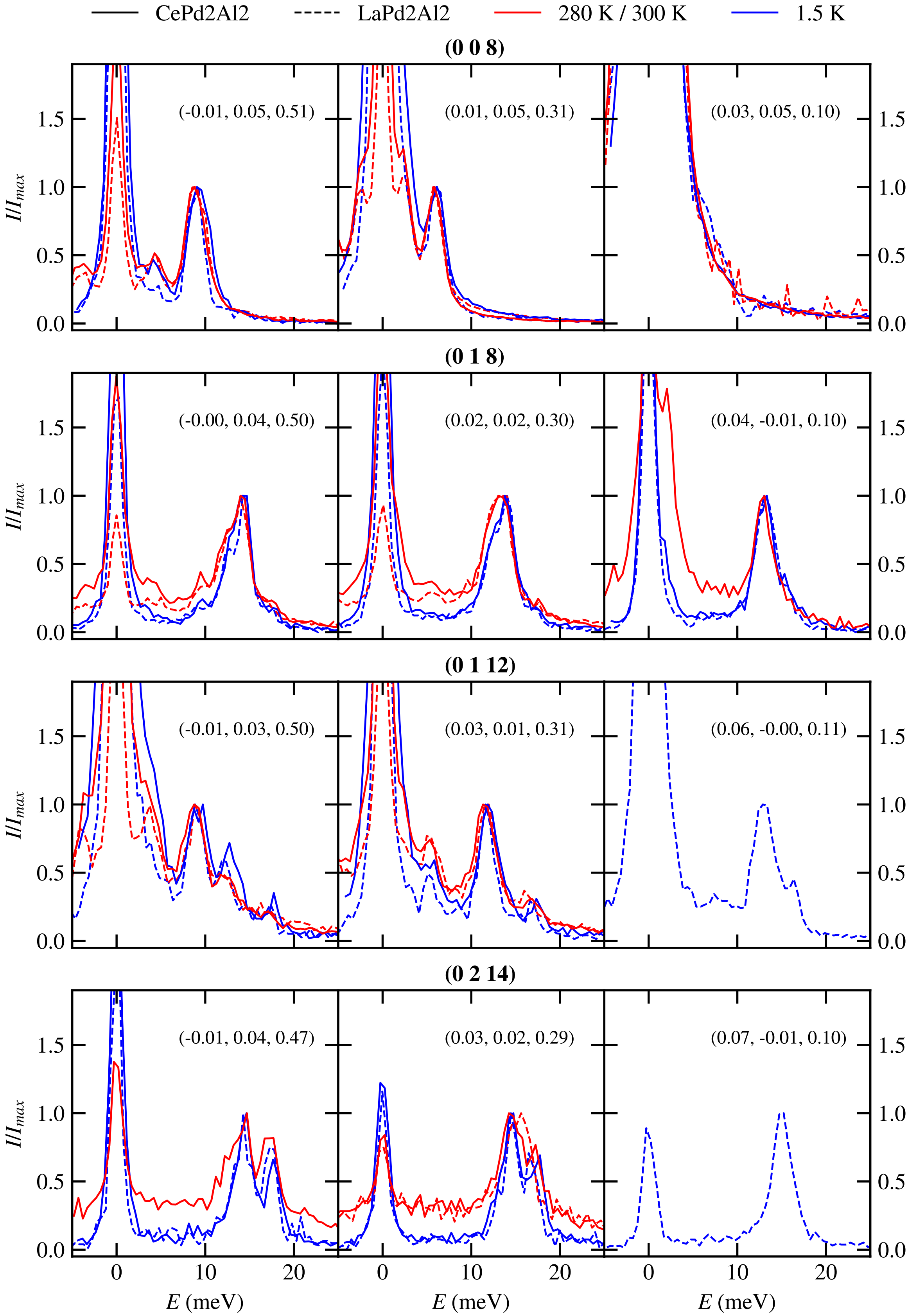

Figure 10: Comparison of measured inelastic X-ray spectra for $CePd_2Al_2$ and $LaPd_2Al_2$ at different Brillouin zones (in bold above the graphs) in the Λ direction. For details, see caption of Fig.6.

Calculated inelastic X-ray spectra in (Ce,La)Pd_2Al_2

For simulation of the expected inelastic X-ray spectra the dynamical structure factor was used, with expression given in [2]. The eigenvectors were calculated in [1]. The profile function was determined by the measurement and corresponds to the experimental resolution.

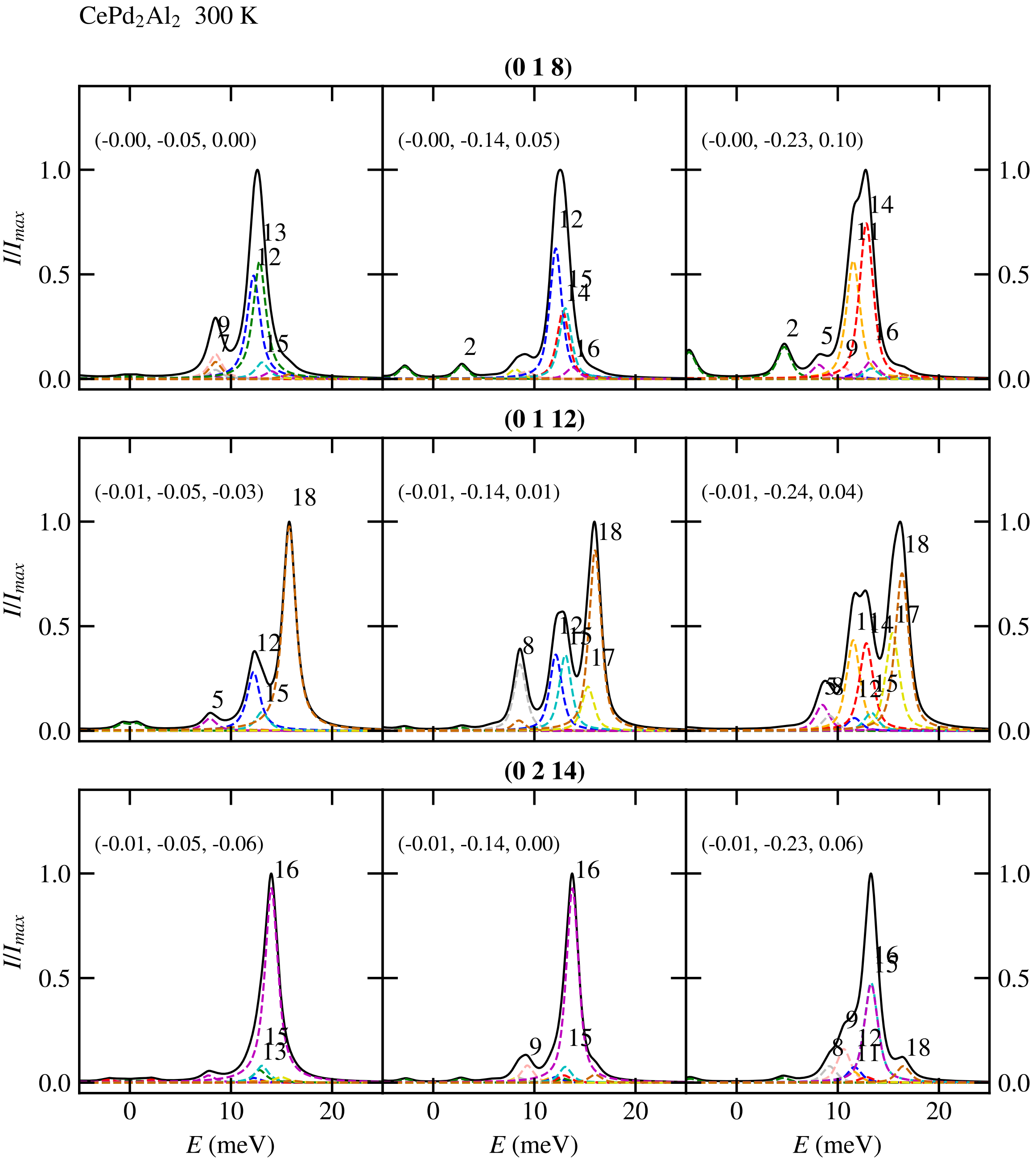

Figure 11: Calculated inelastic X-ray spectra for $CePd_2Al_2$ at different Brillouin zones (in bold above the graphs) at 300 K in the Δ direction - part 1. The position at Brillouin zone is given by the **q** vector with coordinates in brackets. The most intense modes are labelled.

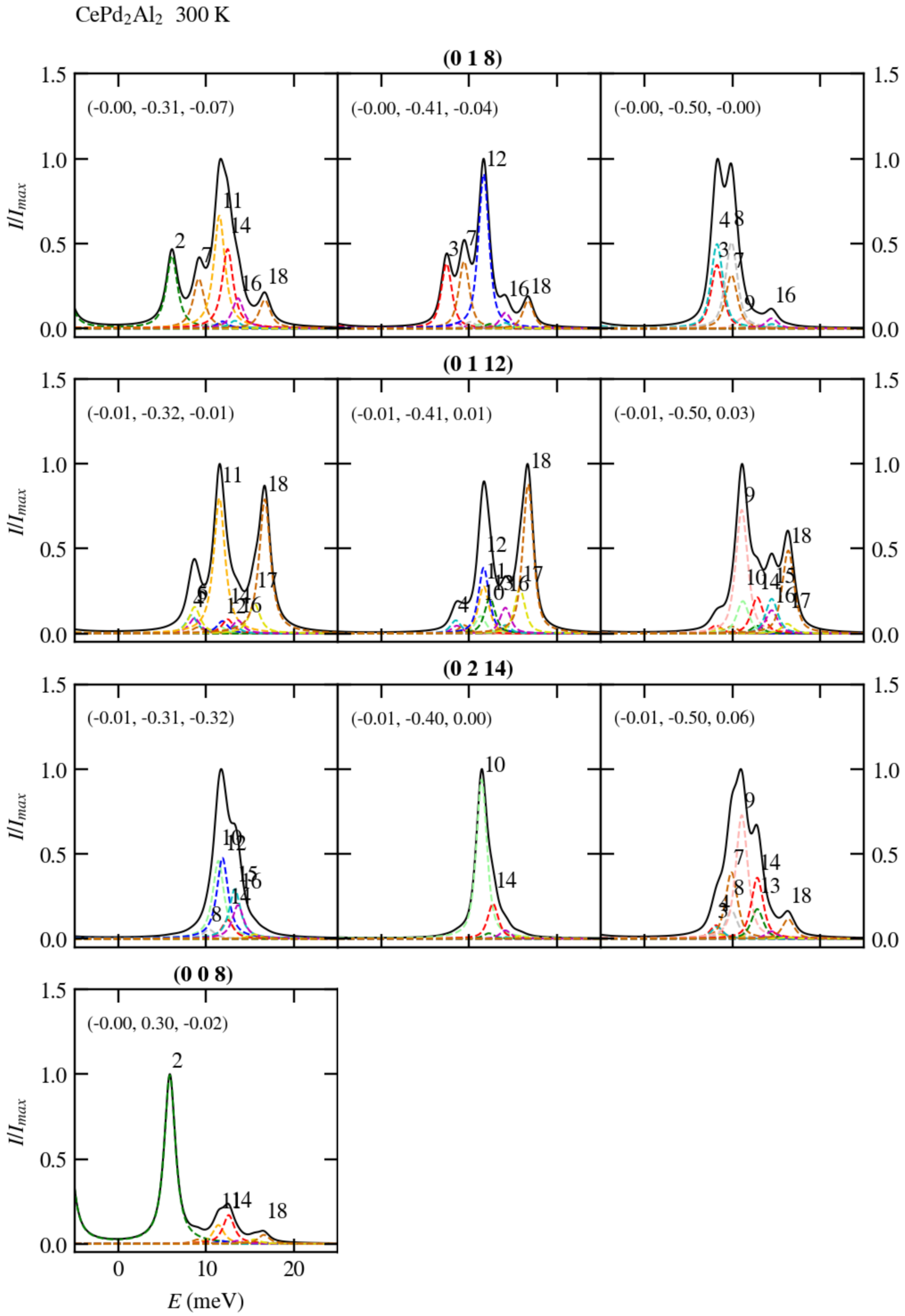

Figure 12: Calculated inelastic X-ray spectra for $CePd_2Al_2$ at different Brillouin zones (in bold above the graphs) at 300 K in the Δ direction - part 2. The position at Brillouin zone is given by **q** vector with coordinates in brackets. The most intense modes are labelled.

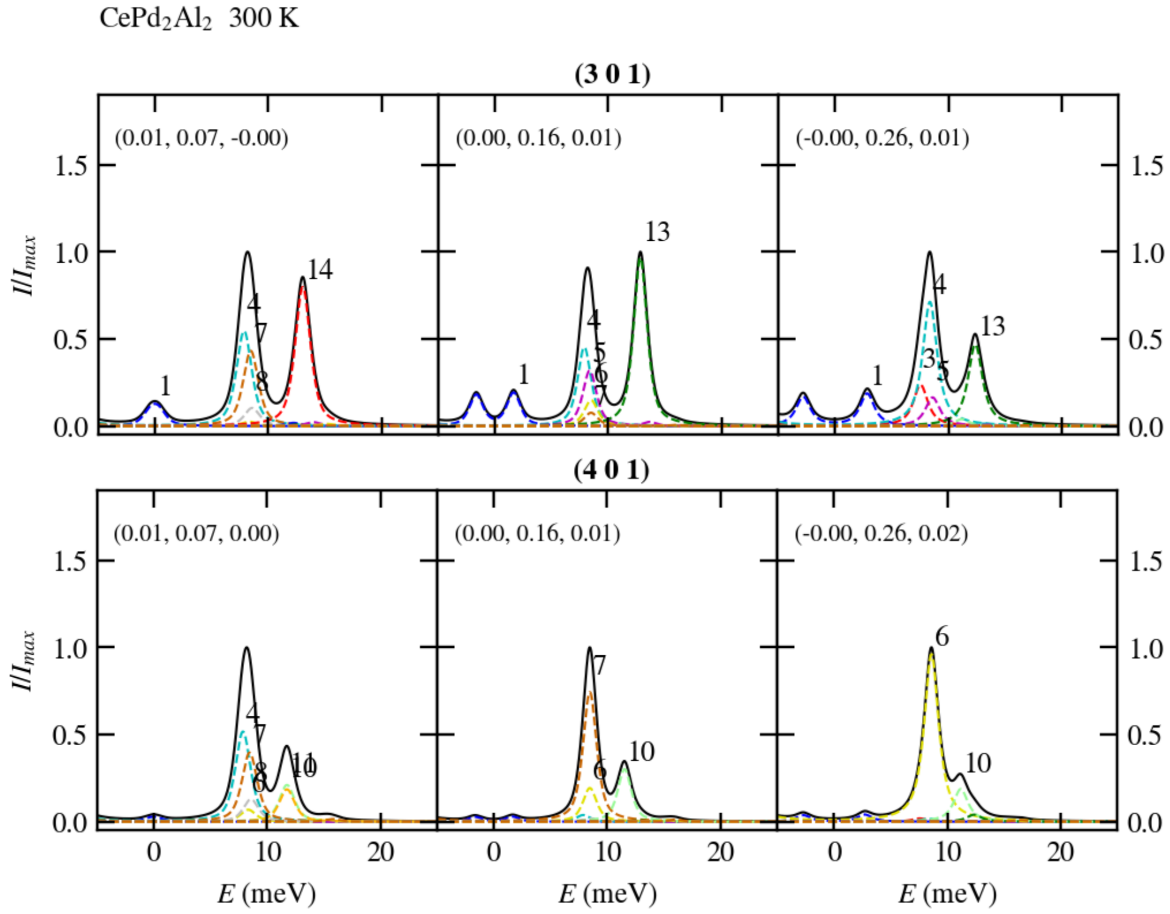

Figure 13: Calculated inelastic X-ray spectra for CePd$_2$Al$_2$ at different Brillouin zones (in bold above the graphs) at 300 K in the Δ direction - part 3. The position at Brillouin zone is given by $\mathbf{q}$ vector with coordinates in brackets. The most intense modes are labelled.

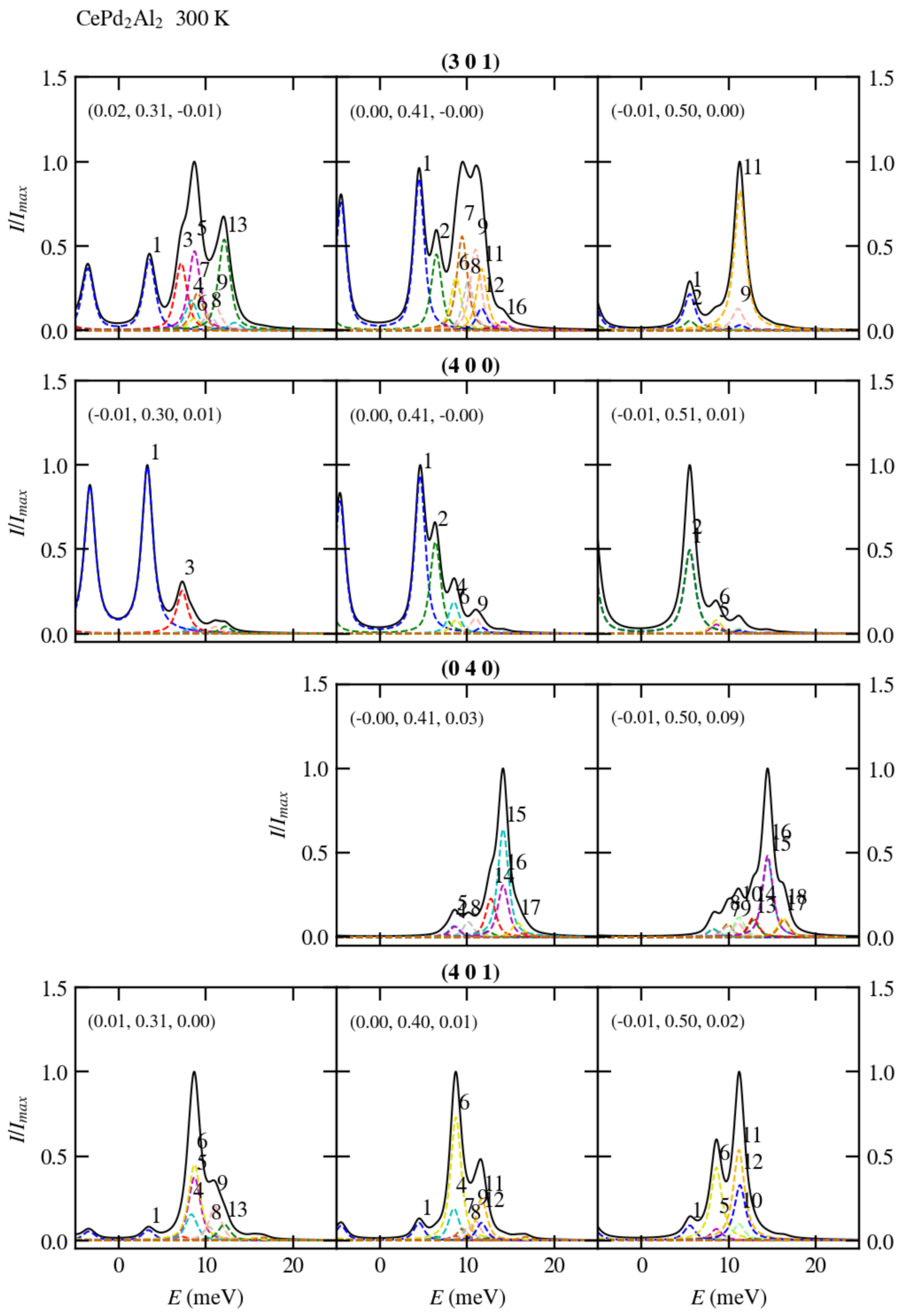

Figure 14: Calculated inelastic X-ray spectra for CePd$_2$Al$_2$ at different Brillouin zones (in bold above the graphs) at 300 K in the Δ direction - part 4. The position at Brillouin zone is given by **q** vector with coordinates in brackets. The most intense modes are labelled.

CePd$_2$Al$_2$ 300 K

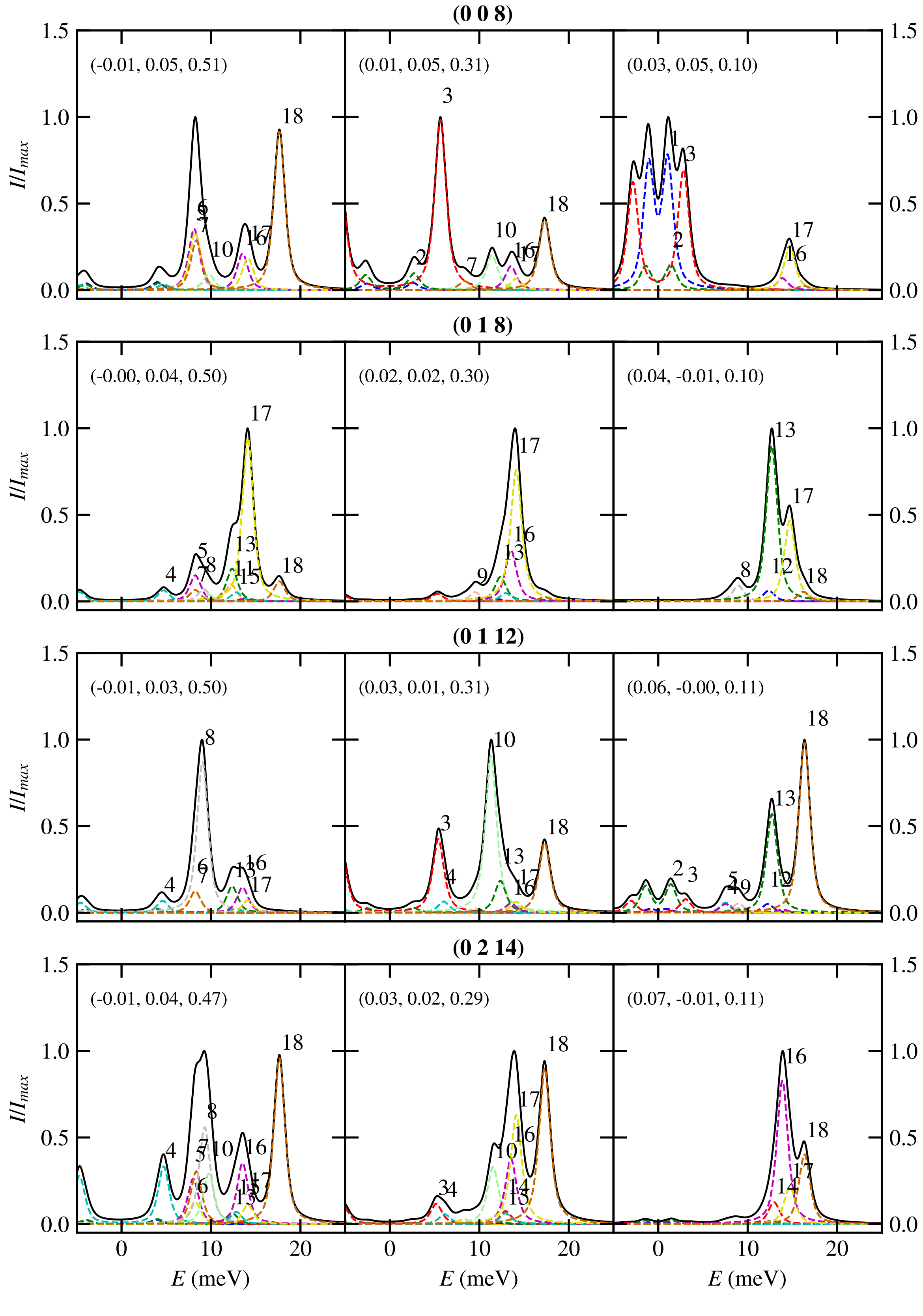

Figure 15: Calculated inelastic X-ray spectra for CePd$_2$Al$_2$ at different Brillouin zones (in bold above the graphs) at 300 K in the Λ direction. The position at Brillouin zone is given by **q** vector with coordinates in brackets. The most intense modes are labelled.

References

[1] D. Legut, M. Diviš, P. Doležal, S. H. Zhang, P. Javorský, Ab initio calculations of the crystal field and phonon dispersions in $CePd_2Al_2$ and $LaPd_2Al_2$, Journal of Physics: Condensed Matter 32 (2020) 235402. doi:10.1088/1361-648X/ab7031.

[2] A. Q. R. Baron, Phonons in Crystals using Inelastic X-Ray Scattering, Journal of The Spectroscopical Society of Japan 58 (5) (2009) 205–2014. doi:arXiv:0910.5764-englishversion.
